# Breast Implant Capsule-Associated Squamous Cell Carcinoma: A Systematic Review and Case Presentation

**DOI:** 10.1007/s00266-023-03693-5

**Published:** 2023-10-05

**Authors:** Danniel Badri, Nicolas Copertino

**Affiliations:** 1 Department of Surgery, Sunshine Coast University Hospital, Main Hospital Building, Level 1, Clinical Department Offices, 6 Doherty Street, Birtinya, QLD 4575 Australia; 2 Faculty of Medicine, The University of Queensland, 288 Herston Road, Brisbane, QLD 4006 Australia

**Keywords:** Breast, Implant, Implant capsule, Squamous cell carcinoma, Systematic review, SCC

## Abstract

**Abstract:**

Breast implant capsule-associated squamous cell carcinoma is exceedingly rare, with only eleven previously published cases. The present study systematically reviews the current literature and describes an additional case in a 56-year-old patient who had undergone previous breast implant augmentation with textured silicone implants 22 years prior. Systematic review of the literature demonstrated a scarcity of reported cases, yielding only eleven other incidences. Symptomatology for this patient involved pain, swelling, and erythema of the right breast prior to eventual surgery. Magnetic resonance imaging reporting extensive oedema and a large periprosthetic effusion with lobulated changes. The patient proceeded with bilateral capsulectomies and histology demonstrated moderately differentiated squamous cell carcinoma, areas of squamous metaplasia, and a chronic inflammatory cell infiltrate. Postoperatively, a positron-emission tomography scan showed no concerning uptake of fluorodeoxyglucose and no evidence of metastatic disease. The patient proceeded to a right-sided total mastectomy and axillary lymph node biopsy. Final histology demonstrated remnant well-differentiated squamous cell carcinoma, whilst five lymph nodes were negative of disease. The patient received postoperative radiation therapy. A clinical history of swelling and pain appears to be a common presentation for this condition. Aspirations of periprosthetic collections containing squamous cells should be considered concerning for neoplasm. The presence of squamous metaplasia within the specimen provides some credence for transformation to invasive carcinoma mediated through chronic inflammation. The presence of perineural invasion would be worth reporting in future cases as it may confer similar risk characteristics as in cutaneous squamous cell carcinoma. A finding of remnant carcinoma during completion mastectomy provides support for an aggressive approach to surgical resection.

**Level of Evidence V:**

This journal requires that authors assign a level of evidence to each article. For a full description of these Evidence-Based Medicine ratings, please refer to the Table of Contents or the online Instructions to Authors www.springer.com/00266.

## Introduction

Breast implant augmentation is one of the most common cosmetic procedures performed by surgeons and is considered to be relatively low risk [1]. However, recently, a rare subset of implant-associated neoplasms have been recognised, highlighted by an increasing incidence of breast implant-associated anaplastic large cell lymphoma [2]. Exceptionally, more scarce in the literature are reports of breast implant capsule-associated squamous cell carcinoma, with only eleven cases published worldwide. The present study systematically reviews the current literature and describes an additional case of breast implant capsule-associated squamous cell carcinoma, in a 56-year-old patient who had undergone previous breast implant augmentation with textured silicone implants.

## Methods

A systematic review was undertaken and reported in accordance with the guidelines in the Preferred Reporting Items for Systematic Reviews and Meta-Analyses (PRISMA) statement [3]. A literature search was performed that aimed to identify any publications, prior to 23 September 2022, which may have reported primary squamous cell carcinoma of a breast implant capsule in patients that had undergone prior breast implant augmentation. The primary author (DB) screened titles and abstracts for relevance, in order to select potentially suitable papers for full-text review. Subsequently, the selected full-text articles were assessed for eligibility, with selected articles additionally being reviewed by the second author (NC).

To identify articles for inclusion in qualitative synthesis, searches were performed in PubMed, Cochrane, EMBASE, and Medline databases for published literature in English. The search terms used were “breast capsule” or “breast implant” or “implant capsule” or “breast augmentation” or “breast implant capsule” and “squamous cell carcinoma”. Manuscripts were included that entailed designs including: randomised control trials, cohort studies (prospective and retrospective), case series, and case reports.

Studies were excluded if patients had undergone non-implant-based breast augmentation, if squamous cell carcinoma originated from breast parenchyma, or if only squamous metaplasia had been reported. Articles that were not accessible online were excluded, noting authors endeavoured to locate all available full-text studies. Only published or accepted manuscripts were included, and other data or abstracts were excluded.

## Results

### Case Report

A 56-year-old woman, with no prior history of breast cancer or skin cancer, underwent bilateral breast implant augmentation with pre-pectoral (macro)textured silicone McGhan implants through periareolar incisions approximately 22 years prior to presentation. The patient had no other past medical history aside from endometriosis and was an active smoker. Symptomatology started for this patient 2 years prior to presentation to the surgical department, with intermittent bilateral breast pain reported. The patient had an ultrasound organised by her primary care physician, which showed bilateral capsular contracture and minimal pericapsular effusions; fine needle aspiration of the effusions was non-diagnostic.

**Fig. 1:**
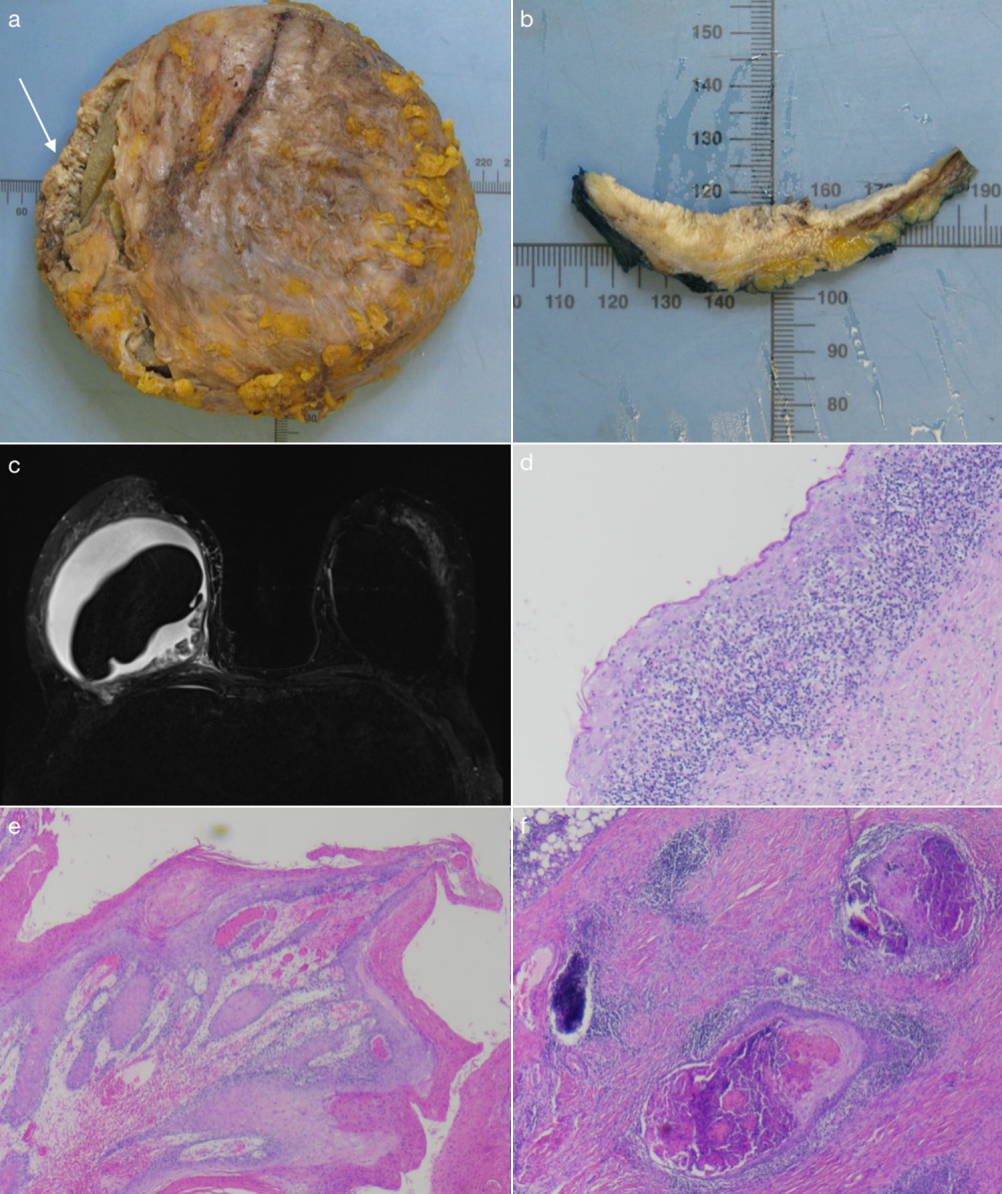
Images from case of breast implant capsule-associated squamous cell carcinoma. **a** Capsulectomy specimen demonstrating fibrotic change and visible verrucous projections of internal surface of implant capsule (arrow). **b** Cross-sectioned sample of capsulectomy specimen demonstrating irregular cream lesion with invasive growth pattern into capsule. **c** Magnetic resonance imaging preoperatively demonstrating periprosthetic collection surrounding a right-sided breast implant, with an irregular lesion lining the posteromedial aspect of the implant capsule. Histological examination of the breast implant capsule showing: **d** squamous metaplasia, **e** transition to invasive squamous cell carcinoma, **f** deeper nests of squamous cell carcinoma

The patient experienced increased pain, swelling, and erythema of the right breast 6 months prior to eventual surgery, with an ultrasound organised through the Emergency Department showing echogenic material surrounding the right implant. A subsequent magnetic resonance imaging scan noted extensive oedema and a large periprosthetic effusion with lobulated changes (Fig. 1c). Analysis of two separate aspirates of thick, turbid fluid from the right breast demonstrated squamous cells but no evidence of anaplastic large cell lymphoma.

Upon presentation, examination revealed Grade IV Baker contracture of a right breast that was grossly swollen and painful to palpation, whilst there was a Grade III Baker contracture of the left breast. The patient proceeded with a bilateral explantation of breast prostheses and bilateral capsulectomies. Intraoperatively, the right capsule was noted to be distorted, fibrotic, with internal verrucous projections of yellow granular material (Fig. 1a, b). Encapsulated within was a chalky/green, viscous periprosthetic collection. The breast implant was intact.

Histology (Fig. 1d–g) demonstrated an irregular cream lesion measuring 100x65x10mm on the internal surface of the capsule, noted to have an infiltrative growth pattern into the capsule and consistent with moderately differentiated squamous cell carcinoma. The capsule was otherwise lined by well-differentiated squamous cell carcinoma, copious keratinisation and papillary projections. Areas of squamous metaplasia and a chronic inflammatory cell infiltrate were noted. There was intraneural invasion (nerve diameter 0.2mm). No carcinoma was seen to extend through the fibrous capsule, and there was no breast parenchymal invasion. Flow cytometry was negative for anaplastic large cell lymphoma, including special testing for a T cell population demonstrating CD30 receptor positivity (CD30 +).

2 weeks postoperatively, a positron-emission tomography scan was performed demonstrating no concerning uptake of fluorodeoxyglucose associated with either breast and no evidence of metastatic disease. The patient proceeded to have a right-sided total mastectomy and axillary lymph node biopsy. Intraoperatively, a small area of residual capsule adherent to the underlying pectoralis major was excised from otherwise preserved tissue planes. Histology demonstrated a single focus of remnant well-differentiated squamous cell carcinoma 0.4 mm in diameter, clear of margins, and remaining breast tissue was unremarkable. Five lymph nodes were sampled and were all negative of disease.

The patient received postoperative radiation therapy of 50 Gy in 25 Fractions. There was no clinical evidence of disease recurrence on examination 4 months after index surgery; a surveillance computed tomography scan was organised for 6 months postoperatively and was reported as negative for local or metastatic recurrence.

### Systematic Review

**Fig. 2:**
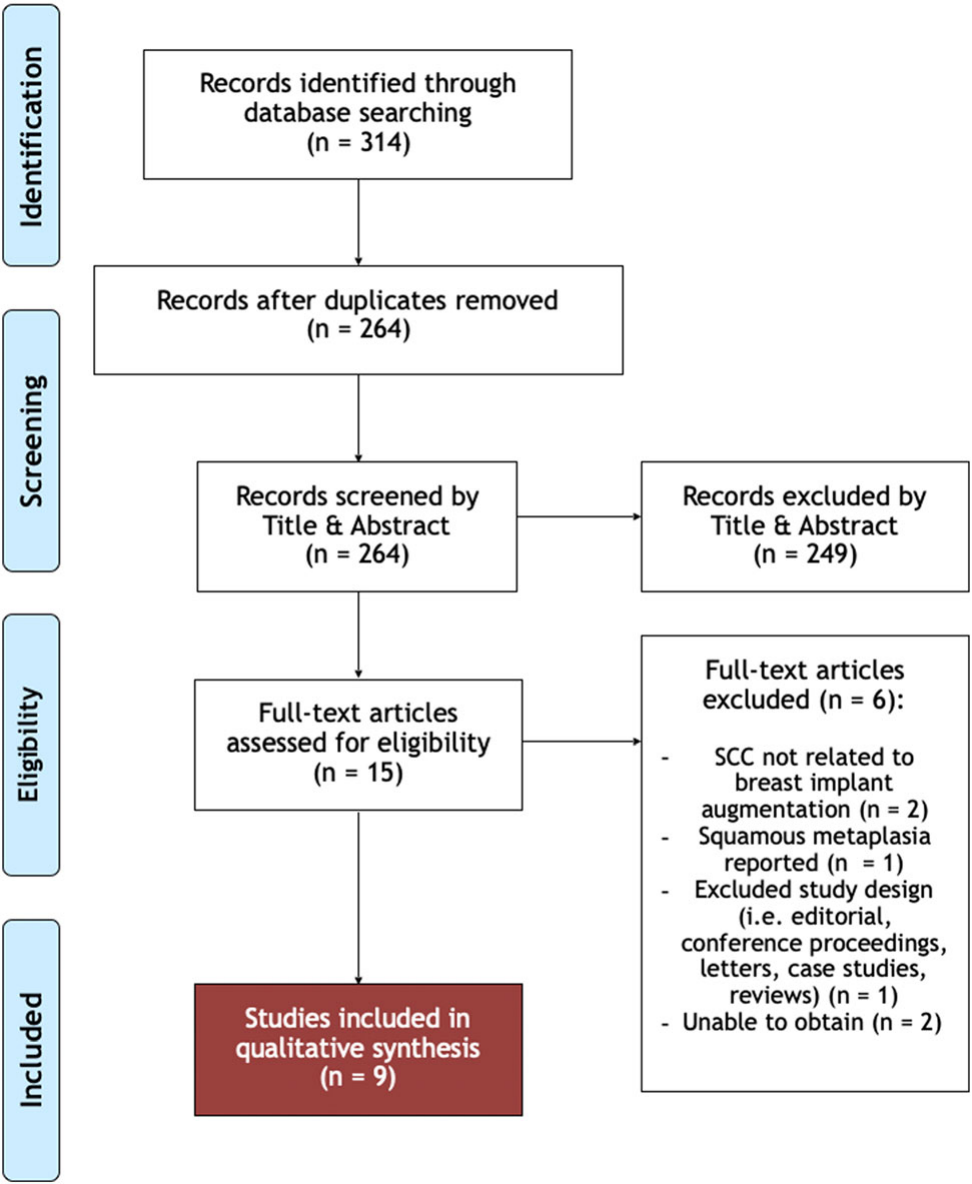
PRISMA flow diagram. Literature search, for breast implant capsule-associated squamous cell carcinoma, undertaken in PubMed, Cochrane, EMBASE, and Medline databases. (From inception to 23rd of September 2023). Modified from Preferred Reporting Items for Systematic Reviews and Meta-Analyses [3]

A total of 314 papers were retrieved, with 264 remaining after removal of duplicates. These were screened using titles and abstracts, selecting 15 papers for full-text review. Nine papers met eligibility criteria and reported a total of eleven cases of breast implant-associated squamous cell carcinoma. A summary of the literature search and exclusions is depicted in Fig. 2.

## Discussion

Presented is an additional patient with breast implant capsule-associated squamous cell carcinoma. The present case is the twelfth reported diagnosis in the English literature to date. The features of the current case and the previously documented patients are summarised in Table 1.

**Table 1:** Review of the literature detailing reported cases of breast implant capsule-associated squamous cell carcinoma

Study	Patient	Age (years)	Presentation	Type of implant	Implant status	Time from augment to diagnosis (years)	Therapeutic treatment	Tumour differentiation	Tumour size	Depth of invasion	Nodal involvement	Metastases (at time of diagnosis)	Follow-up
Paletta et al. [4], Kitchen et al. [5]	1	52	Enlargement, pain	Silicone (Heyer Schulte)	Intact	15	Bilateral breast implant removal and capsulectomy, subsequent left modified mastectomy and axillary lymph node dissection	Poorly differentiated, sarcomatoid differentiation	6cm	Intracapsular	None	None	No evidence of disease at 12 months
Zomerlei et al. [6]	2	58	Painful swelling, erythema, cutaneous thinning	Smooth, silicone †	Intact	15	Breast implant removal, subsequent right total mastectomy and sentinel lymph node biopsy (with concurrent left explant and simple mastectomy)	Moderately differentiated	5.5cm	NR	0/30 Lymph nodes	None	NR
Olsen et al. [7]	3	56	Enlargement, pain, red-purple skin discolouration	Textured saline †	Intact	28	Breast implant removal, Subsequent left mastectomy and Sentinel lymph node biopsy, Adjuvant Chemotherapy & Radiotherapy	Well- to Moderately differentiated	3.5cm	Extracapsular extension, into breast parenchyma and chest wall skeletal muscle	0/9 Lymph nodes	NR	Metastatic disease in left axilla, left upper arm, and left chest wall at 8 months
	4	81	Enlargement, pain, palpable breast mass	Silicone	Intact	42	Breast implant removal, subsequent left mastectomy and Sentinel lymph node biopsy	Moderately differentiated, sarcomatoid/spindle cell differentiation	5cm	Extracapsular extension, into breast parenchyma	0/3 Lymph nodes	NR	Metastatic disease in lung, liver, soft tissue of leg, and mediastinum/hilum at 5 months. Subsequently died of disease
Zhou et al. [8]	5	46	Swelling, hardening	Silicone†	Intact	19	Bilateral breast implant removal and capsulectomy, subsequent re-excision of remnant chest wall mass, adjuvant radiotherapy	Moderately differentiated	4cm	Extracapsular extension, into skeletal muscle	–	None	Died with metastatic disease at 17 months
Buchanan et al. [9]	6	65	Enlargement, pain	Foam covered silicone (Heyer Schulte)	Ruptured	35	Capsulectomy and implant exchange, subsequent left radical mastectomy and medial chest wall resection, Adjuvant Radiotherapy	Well-differentiated	NR	Extracapsular extension, into pectoralis major	–	PET avid axillary and internal mammary lymph nodes	No evidence of disease at 96 months
Goldberg et al. [10]	7	40	Swelling, erythema, clear nipple discharge	Smooth, saline	Intact	11	Bilateral breast implant removal and capsulectomy, neoadjuvant chemotherapy whilst planning further resection	Moderately differentiated	NR	Extracapsular extension, into pectoralis minor & chest wall	–	None	Died with malignant pleural effusions at 3 months
	8	62	Painful swelling, erythema	Smooth, silicone	Intact	32	Bilateral breast implant removal and capsulectomy	Well-differentiated	NR	Intracapsular	–	None	Lost to follow-up
Whaley et al. [1]	9	60	Painful swelling, erythema, cutaneous necrosis, serous discharge	Textured saline	Ruptured	27	Bilateral breast implant removal and capsulectomy	Well-differentiated	3cm	Intracapsular	–	None ‡	No evidence of disease at 9 months
	10	57	Painful swelling, erythema, purulent discharge	Saline	NR	25	Capsulectomy, subsequent mastectomy	Well-differentiated	7.5cm	Extracapsular extension, but no parenchymal invasion	–	None ‡	Lost to follow-up
Soni et al. [11]	11	46	Painful swelling	Smooth, round, saline (Mentor) †	Intact	NR	Modified radical mastectomy and sentinel lymph node biopsy, adjuvant chemotherapy & Radiotherapy	Well-differentiated	6cm	Breast parenchyma & skeletal muscle	None	NR	No evidence of disease at 12 months
Present study (2023)	12	56	Painful swelling	Textured, silicone (McGhan)	Intact	22	Capsulectomy, subsequent mastectomy and axillary lymph node biopsy	Moderately differentiated	10cm	Intracapsular	0/5 Lymph nodes	None	No evidence of disease at 6 months

NR, Not Reported

^†^Patient was reported to have previous revision surgery or alternate implants

^‡^Study did not detail investigations used to determine this finding

Comparable to the presenting age (average of 56.6 years) of other patients with breast implant capsule-associated squamous cell carcinoma, this patient was diagnosed at 56 years old. Furthermore, the diagnosis was made 22 years following breast implant augmentation, congruent with the published average time to presentation of 24.9 years. The implant was a textured silicone implant, with previous implants reported to be textured in 27.2%, smooth in 36.4%, and containing silicone in 54.5%, of cases. Implant details were not readily available in all of the published literature, with the type of shell remaining unreported in 36.4% of the selected articles. The present clinical history included swelling and pain, noted in the literature to occur in 100% and 81.8% of patients, respectively [1, 4–11].

Initial work up of the patient with magnetic resonance imaging was considered concerning for breast implant-associated anaplastic large cell lymphoma, due to the presence of a large periprosthetic lobulated effusion. Whilst anaplastic large cell lymphoma is the most commonly encountered implant-associated neoplastic process, it remains exceedingly rare in its own right [2]. Imaging findings for this condition similarly commonly encompass serous effusions, peri-implant or capsule related masses, and irregular enhancement or thickening of the implant capsule [12].

The most common presentation of anaplastic large cell lymphoma is persistent late periprosthetic effusion or seroma, similar symptomatology to the present clinical history [12, 13]. However, aspiration and flow cytometry proved inconclusive for this diagnosis during diagnostic work up. In retrospect, squamous cells were noted in aspirates (initially considered contaminants) and should not normally reside within the non pathologic breast capsule [1]. Future consideration should be given to aspirates containing squamous cells in patients with imaging concerning for neoplastic transformation.

Postulated in the literature is that chronic inflammation leads to epithelialisation of the internal lining of breast implant capsules, with subsequent metaplasia and, finally, neoplastic transformation of these cells to squamous cell carcinoma [14]. Notably, in the current breast implant capsule specimen, a chronic cellular infiltrate and areas of squamous metaplasia were observed microscopically. Interestingly, 80% of adequately reported cases described an intact breast implant, contrary to hypotheses in the literature of rupture as a causative event in promoting cellular inflammation and metaplasia [14].

The pathogenesis of anaplastic large cell lymphoma is still incompletely understood, but is thought to be a complex process that includes textured implant surfaces predisposing to bacterial biofilm formation, which in turn leads to immune system activation and chronic inflammation, a known predisposition for lymphoma development [2]. A future direction of research could be investigation of the mechanisms that predispose to the formation of breast implant-associated squamous cell carcinoma over anaplastic large cell lymphoma. The meticulous reporting of future cases, and analysis of pathologic capsules, will be important in compiling evidence to explain this phenomenon.

Histological analysis of the caspulectomy specimen demonstrated moderately differentiated squamous cell carcinoma, measuring 100 mm at maximum extent, notable as this would be the largest reported lesion in the literature [1, 4–11]. Infiltration into the breast capsule was visualised although it remained intracapsular, potentially a prognostic feature for future analyses, as in reported intracapsular cases (including the present) there was no evidence of disease in patients attending follow-up (average time of follow-up 8.3 months) [1, 4, 5]. A limitation of the current paper is that the presented case was only followed up with clinical examination and surveillance scanning for 6 months postoperatively thus far.

There was intraneural invasion of nerve 0.2 mm in diameter. In cutaneous squamous cell carcinoma, the presence of neural invasion in nerves >0.1 mm carries an increased risk of metastases, recurrence, and poor prognosis [15]. The presence of this histopathological finding remains of uncertain significance at present due to the scarcity of cases, but would be worth reporting in future cases as it may confer similar risk characteristics as in cutaneous lesions.

As aforementioned, during the subsequent right-sided total mastectomy, a single focus of remnant squamous cell carcinoma 0.4mm in diameter was discovered. Previous authors have proceeded to mastectomy following the diagnosis of squamous cell carcinoma in 63.6% of patients [1, 4–7, 9, 11]. Whilst there remains a scarcity of evidence, the finding of remnant carcinoma (as well as the reported aggressive nature of previous cases) suggests completion mastectomy should be considered following diagnosis of breast implant capsule-associated squamous cell carcinoma.

## Conclusion

In summary, presented is an additional patient with breast implant capsule-associated squamous cell carcinoma. A systematic review of the literature demonstrated a rarity of reported cases, yielding only eleven other incidences. The clinical, radiological, and histopathological findings are discussed. Elucidated are potentially important diagnostic features whilst pathogenetic factors and treatment recommendations are stipulated.
